# Comprehensive Mapping of the Cell Response to *Borrelia bavariensis* in the Brain Microvascular Endothelial Cells *in vitro* Using RNA-Seq

**DOI:** 10.3389/fmicb.2021.760627

**Published:** 2021-11-08

**Authors:** Zuzana Tkáčová, Katarína Bhide, Evelina Mochnáčová, Patrícia Petroušková, Jana Hruškovicová, Amod Kulkarni, Mangesh Bhide

**Affiliations:** ^1^Laboratory of Biomedical Microbiology and Immunology, The University of Veterinary Medicine and Pharmacy, Kosice, Slovakia; ^2^Institute of Neuroimmunology, Slovak Academy of Sciences, Bratislava, Slovakia

**Keywords:** *Borrelia bavariensis*, BBB, transcriptom, RNA-seq, endothelial cells, BBB crossing

## Abstract

*Borrelia bavariensis* can invade the central nervous system (CNS) by crossing the blood-brain barrier (BBB). It is predicted that *B. bavariensis* evokes numerous signaling cascades in the human brain microvascular endothelial cells (hBMECs) and exploits them to traverse across the BBB. The complete picture of signaling events in hBMECs induced by *B. bavariensis* remains uncovered. Using RNA sequencing, we mapped 11,398 genes and identified 295 differentially expressed genes (DEGs, 251 upregulated genes and 44 downregulated genes) in *B. bavariensis* challenged hBMECs. The results obtained from RNA-seq were validated with qPCR. Gene ontology analysis revealed the participation of DEGs in a number of biological processes like cell communication, organization of the extracellular matrix, vesicle-mediated transport, cell response triggered by pattern recognition receptors, antigen processing via MHC class I, cellular stress, metabolism, signal transduction, etc. The expression of several non-protein coding genes was also evoked. In this manuscript, we discuss in detail the correlation between several signaling cascades elicited and the translocation of BBB by *B. bavariensis*. The data revealed here may contribute to a better understanding of the mechanisms employed by *B. bavariensis* to cross the BBB.

## Introduction

Lyme borreliosis is the most common tick-borne disease in Europe caused by bacteria of the *Borrelia burgdorferi* sensu lato (B.b.s.l.) complex, in Europe and Asia predominantly by *B. afzelii, B. bavariensis*, and *B. garinii* (Barbour and Fish, 1993; Stanek et al., 2012; Thompson et al., 2020b). The initial stages of Lyme borreliosis are characterized by the presence of erythema migrans (Thompson et al., 2020b), headache, fatigue, or fever (Murray and Shapiro, 2010). If untreated, the members of B.b.s.l. complex in later stages may disseminate into tissues including joints, heart, and central nervous system (CNS) (Biesiada et al., 2012; Rauer et al., 2020).

Some members of B.b.s.l., like *B. garinii* and *B. bavariensis*, can invade the CNS by crossing the blood-brain barrier (BBB) mainly via a paracellular route (crossing of pathogens through intercellular space) (Szczepanski et al., 1990; Grab et al., 2005), however, a possible transcellular mechanism (transport through cells) was also demonstrated (Comstock and Thomas, 1989). The BBB is a semipermeable membrane formed by the human brain microvascular endothelial cells (hBMECs) that line the cerebral microvessels from the luminal side (Abbott et al., 2006). The uniqueness of the semipermeable BBB is primarily determined by the presence of endothelial junctional complexes made up of adherens junctions and highly specialized tight junctions (Pulzova et al., 2009). This semipermiability tightly regulates the entry of macromolecules, blood cells, and pathogens from blood circulation into the CNS. *Borrelia*, however, can induce inflammatory responses (production of cytokines IL6, TNFα, and IL1) and activate signaling cascades leading to the proteolysis of intercellular junctions and alter the cell cytoskeletal structure to facilitate BBB traversal (Rupprecht et al., 2008; Pulzova et al., 2009; Bencurova et al., 2011). During the paracellular passage, *Borrelia* induces the host fibrinolytic system and the expression of matrix metalloproteinases (MMPs) in hBMECs to cause focal and transient degradation of tight junction proteins (Grab et al., 2005) and digestion of components of extracellular matrices (ECM) (Klempner et al., 1995; Coleman et al., 1999). Using real-time PCR, genes encoding adhesion molecules (like ICAM-1, VCAM-1, and E-selectin) (Sellati et al., 1995; Wooten et al., 1996), transcription factors (e.g., NF-κB) (Pulzova et al., 2011), metalloproteinases (mostly MMP-3 and MMP-9) (Déchanet et al., 1997; Pulzova et al., 2011), pro-inflammatory cytokines (IL6, TNFα, IL1), and molecules involved in remodulation of cell cytoskeleton (Maruo et al., 1992; Madge and Pober, 2001; Pulzova et al., 2011) were reported to be significantly upregulated in hBMECs upon stationary adhesion of *Borrelia*. Although several reports are available, which show the interaction between *Borrelia* and endothelial cells of the brain microvasculature, results are scattered and obtained through low-throughput techniques.

In the last decade, high-throughput methods like microarray and RNA-seq are commonly used to understand the transcriptomic response of host cells to the various pathogens (Káňová et al., 2019; Kusmierek et al., 2019; Grønnemose et al., 2021). RNA-seq has surpassed microarray in detecting and quantifying transcriptome-wide gene expression and mRNA splicing (Stark et al., 2019). Global transcriptome analysis by RNA-seq on astrocytes (Casselli et al., 2017), epithelial cells (Thompson et al., 2020a), and monocytes (Salazar et al., 2009) infected with *B. burgdorferi* has revealed possible involvement of receptors (e.g., TLR2, TLR1, CD14) (Salazar et al., 2009), proteases (MMPs and plasminogen), and host inflammatory molecules (TNFα, IL6, IL10, and IL1β) in pathogenesis.

To our knowledge, no study is available that presents a complete picture of the signaling events evoked in endothelial cells of the brain microvasculature by borrelial infection. Thus, in this study, we mainly aimed to unfold changes in the gene expression involved in a variety of molecular processes that help *Borrelia* to cross BBB via the paracellular route, namely, cell surface modification upon adhesion of *Borrelia* on hBMECs, regulation of junctional proteins, and reorganization of extracellular matrix including plausible involvement of host proteases. Further, it was interesting to find *B. bavariensis* deregulates genes involved in the host cell metabolism and cellular stress, and how it evokes expression of non-coding RNAs in hBMECs. Finally, we have found the expression of genes involved in innate immune responses. Although the endothelial cells are not part of the immune system, many immune related genes are activated by *Borrelia*. As the activation of immune pathways like NF-κB or TNF leads to upregulation of adhesion molecules (e.g., ICAM-1 and VCAM-1) and recruitment of leukocytes at the site of infection, this may ultimately help *Borrelia* to adhere to endothelial cells and cross the BBB. Therefore, knowledge of the expression of genes related to the immune system is important. The results presented in this paper will help to understand molecular mechanisms employed by *B. bavariensis* to cross the BBB.

## Materials and Methods

### Culture of Human Brain Microvascular Endothelial Cells

Human BMECs (Merck Millipore, Czech Republic) were cultured as described previously (Jiménez-Munguía et al., 2018). Human BMECs (hBMEC/D3 cells) were purchased from Merck/Millipore (Prague, Czech Republic). Cells were cultured in a 25-mL cell culture flask coated with collagen type I (Sigma, United States) in EBM-2 medium (Lonza, United Kingdom) containing 10% FBS, gentamycin, 5 μg/mL ascorbic acid, 10 mM HEPES, 1.4 μM hydrocortisone (Sigma) and 1 ng/mL bFGF (Sigma). Cells were incubated at 37°C in a humid atmosphere of 5% CO_2_ until confluence. Cells from the monolayer (6th passage) were seeded in 6-well plates for incubation with *B. bavariensis.*

### *Borrelia bavariensis* Culture

*Borrelia bavariensis* (strain SKT7.1), a neuroinvasive strain (Margos et al., 2013; Di Domenico et al., 2018; Sertour et al., 2018), was grown in complete BSK-II medium enriched with 6% rabbit serum at 34°C (Sigma, United States). Please note that neuroinvasive *B. garinii* serotype IV was renamed as *B. bavariensis* as per the revised nomenclature (Margos et al., 2009). The culture was passaged in BSK-II medium (pH 6.8) enriched with 6% human serum (Sigma, inactivated at 56°C), and incubated at 35°C for 5 days to induce surface proteins that are expressed in mammalian host environment. After 2 weeks, cultures were examined under the dark field microscope to assess the borrelial viability and motility.

Before induction of the cells, *B. bavariensis* were washed with minimal essential medium (Biowest, France), and the concentration was set to 4 × 10^5 *Borrelia*/mL. *Borrelia bavariensis* were enumerated using flow cytometry (BD Accuri C6, United States). Events were acquired in the list mode (10 μL/min). The sample flow rate was kept slow and the core size was set to10 μm. *Borrelia* spirochetes were differentiated from any debris or particles from the minimal essential medium by adjusting the threshold of forward scatter (FSC-H 80,000). Non-rectangular gate in the dot plot [side-scatter (SSC-A) vs. forward scatter (FSC-A)] was defined to enumerate spirochetes in the sample.

### Challenge of Human Brain Microvascular Endothelial Cells

hBMECs were cultured in 6-well plates until 75% confluency. Cells were incubated either with live *B. bavariensis* (MOI 0.5) or just the culture medium (mock control) for 6 h at 37°C. The experiments were performed in triplicate. mRNA from hBMECs was isolated using RNeasy Mini Kit (Qiagen, Germany) according to the manufacturer’s instructions. DNaseI (Qiagen) treatment was incorporated during RNA isolation. The RNA concentration and purity were measured using NanoDrop (Thermo Fischer Scientific). The quality of RNA was checked using capillary electrophoresis (Fragment analyzer, Advanced Analytical Technologies, Inc., United States).

### Preparation of cDNA Library and NGS Sequencing

cDNA library preparation, quality control of the library, NGS sequencing, and data analysis were performed exactly as described in our recent publication (Káňová et al., 2019). All steps were performed as per the manufacturer’s instructions (QuantSeq 3′ mRNA-Seq Library Prep Kit; Lexogen, Austria). There was 250 ng of RNA reverse transcribed with oligod T primers and the first strand of cDNA was synthesized. RNA template was removed (RNA removal solution, RS buffer) and the second strand was synthesized using random hexamer primer that contains Illumina-compatible linker sequences at its 5′ end. Double-stranded DNA libraries were purified using magnetic beads. Each library was amplified by PCR using unique single indexing i7 primers to add the complete adapter sequence required for cluster generation. The number of cycles in PCR for each library was determined using the qPCR add-on kit from Illumina (Lexogen). Cycles used for library amplification were as follows: hBMECs induced with *Borrelia*—20 cycles, non-induced cells—17 cycles. Please note that qPCR-based add-on kit is necessary to determine the exact number of cycles for the PCR with i7 indexing primers in order to prevent any under- or overcycling of a library for sequencing. Undercycling may result in too little library while overcycling can lead to significant distortions in gene expression values.

Amplified libraries were purified using magnetic beads, the quality of the libraries, and the length of the fragments were checked on the fragment analyzer. Libraries were sequenced on Illumina NextSeq, single-end 75 bp, to a minimum depth of 8 million reads per sample. Fastq files were processed and aligned to the reference genome (*Homo sapiens* GRCh38) using the STAR aligner. Gene counts were generated using the STAR aligner. R package edgeR was used to analyze differential gene expression. A Venn diagram was constructed to analyze the logical relation of DEGs (differentially expressed genes) between the challenged and non-induced hBMECs.

### Data Analysis

The raw RNA-seq data were deposited into the EBI Arrayexpress repository^1^ under the accession number E-MTAB-8053. Functional analysis of DEGs was performed by the Reactome server^2^ (Croft et al., 2011). The Heatmapper server was used for graphical representation of the DEGs.^3^ Signaling pathways related to the translocation across the BBB (ECM-receptor interaction, focal adhesion, Toll-like receptor signaling pathway, NF-κB signaling pathway, and TNF signaling pathway) were downloaded from the KEEG server^4^ and the DEGs involved in the pathways were manually highlighted.

### Validation of Differentially Expressed Genes by qPCR

The RNA was reverse transcribed using random hexamers (Thermo Fischer Scientific, United States). RNA (1 μg) and random hexamers (100 pMol) were mixed and incubated for 5 min at 65°C. Later, 4 μL of 5x reaction buffer, 1 μL RevertAid reverse transcriptase (200 U), 2 μL dNTP (10 mM), and 0.5 μL RiboLock RNase inhibitor (20 U) (all reagents from Thermo Fisher Scientific) were added and incubated for 10 min at 25°C followed by 1 h at 42°C and 70°C for 10 min.

For validation, a total of 31 genes were selected (20 upregulated and 11 downregulated, Supplementary Data Sheet 1 and Supplementary Table 1) based on the level of expression and involvement in the biological function. For example, we tried to select cell adhesion molecules, interleukins, chemokines, cathepsin, metallopeptidases, and CD molecules. Primers were designed (Geneious 9.1.5 software, Biomatters, United States) to have a melting temperature of around 60°C, with no predicted hairpins and dimers. Care was taken to encompass at least one intron between sense and anti-sense primer (intron spanning). Primer efficiency was calculated as described by the primer manufacturer’s instructions (Sigma), while the specificity of amplification was determined based on high-resolution melting analysis in StepOnePlus thermocycler (Thermo Fisher Scientific). The best 10 primer pairs (6 upregulated and 4 downregulated) were selected for validation with qPCR (Supplementary Data Sheet 1 and Supplementary Table 2). The reaction mix used for qPCR contained 1x qPCR GreenMaster with high ROX (Jena Bioscience, Germany), gene-specific primers (10 pMol each), RNase free water up to a total volume of 20 μL and 6 ng of cDNA. Amplification conditions were maintained as follows: 95°C—10 min, 40x [95°C—15 s, 50—60°C—30 s (annealing temperature varied as per the primers used), 72°C for 30 s (signal capture)], melting curve 60°C to 95°C—0.3% temperature increment/sec (StepOnePlus). qPCR was performed in three biological replicates.

Fold expression was calculated taking into account Ct values from mock control (non-induced hBMECs), while Ct values of β–2-microglobulin (housekeeping gene) were used for normalization. ΔΔCt was calculated as described before (Pulzova et al., 2011) and values were converted to logFC.^5^ Pearson correlation coefficient PCCs were used to correlate expression values obtained from RNA-seq and quantitative PCR.^6^

## Results

### RNA-Seq on Human Brain Microvascular Endothelial Cells Induced With *Borrelia bavariensis*

RNA from all biological replicates used for RNA-seq showed good integrity as the ratio between 28S and 18S RNA was 2:1 (Supplementary Data Sheet 1 and Supplementary Figure 1). The RNA quality number (RQN) was between 7.2 and 10, indicating the suitability of RNA for downstream NGS application. cDNA fragment size in all libraries was between 150 and 350 nt (Supplementary Data Sheet 1 and Supplementary Figure 2). The average number of raw reads obtained from NGS was 13 million in the case of hBMECs incubated with *B. bavariensis*, whereas in the case of the mock control it was 10 million (Supplementary Data Sheet 1 and Supplementary Table 3). There were 11,398 genes mapped for each sample (Supplementary Data Sheet 2). A minimum average logCPM (count per million) 3 and logFC (fold change) more than ± 1.2 were applied to designate a gene as differentially expressed. The differentially expressed genes (DEGs) with *P*-value *P* < 0.01 were included for further analysis (Supplementary Data Sheet 1 and Supplementary Figure 3).

### Validation of the RNA-Seq and Segregation of Differentially Expressed Genes According to the Gene Ontology Biological Processes

**FIGURE 1:**
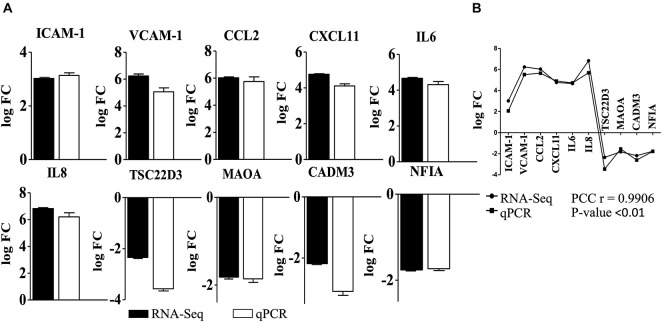
Validation of differentially expressed genes detected in RNA-seq with qPCR. **(A)** Gene expression (logFC) of DEGs used to validate RNA-seq analysis of endothelial cells incubated with *B. bavariensis*. Black bars—logFC values from RNA-seq, white bars—log FC calculated from qPCR. Pearson correlation coefficient (PCC) *r* = 0.9906; *P*-value < 0.01. **(B)** Correlation of the gene expression (log FC) of DEGs obtained from RNA-seq and qPCR after incubation of hBMECs with *B. bavariesis.*

Overall 295 DEGs (251 upregulated and 44 downregulated) were identified in the cells induced with *B. bavariensis* (Supplementary Data Sheet 3) compared to controls. Results of RNA-seq were validated with qPCR. Among 31 primer pairs designed for qPCR, 13 pairs showed high amplification efficiency (≥95%), while 6 pairs showed non-specific amplification judged by high-resolution melting analysis. Ten sets of primers (efficiency more than 97%) were finally used for validation of RNA-seq results. A positive correlation between gene expression values from RNA-seq and qPCR (Pearson correlation coefficient, PCC *r* = 0.9906; *P* < 0.01, Figure 1) was observed. Next, the DEGs were segregated according to the gene ontology (GO) biological processes using the Reactome server (see text footnote 2) (Supplementary Data Sheet 4). In general, the DEGs were enriched in the biological processes, namely, cell-cell communication, cell junction organization, degradation of ECM, laminin interactions, immune response, vesicle-mediated transport, and metabolism. Among these GO biological processes, we focused on genes involved in cell signaling events that are associated with the translocation of the pathogens across the BBB and immune response. Results of these cell signaling events are presented below in detail. As scanty information is available in the literature, which presents changes in the biological processes like cellular metabolism, cellular stress, and signal transduction occurred due to the borrelial infection, we have also included those processes below.

### Differentially Expressed Genes Related to the Cell Communication

**FIGURE 2:**
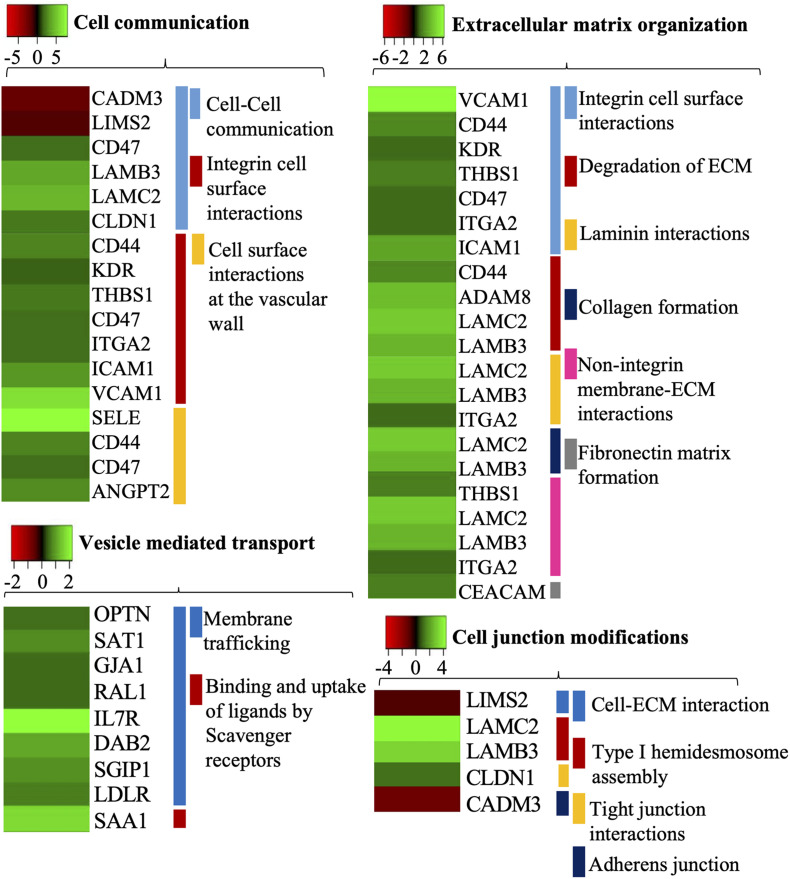
Gene ontology biological processes (cell communication, cell junction modifications, extracellular matrix organization, and vesicle-mediated transport) activated by *B. bavariensis* in hBMECs. Green shaded genes—upregulated, red shaded genes—downregulated. The intensity of the color indicates the degree of expression level. The range of the fold change (logFC) is presented in the scale.

Three GO biological processes, namely, “cell-cell communication,” “integrin cell surface interactions,” and “cell surface interactions at the vascular wall,” related to the cell surface modification can be found in the Reactome database. E-selectin (logFC 7.94) was the most upregulated gene among these biological processes induced by *B. bavariensis* followed by VCAM-1 (logFC 6.23) (Figure 2 and Supplementary Data Sheet 3). CD44, CD47, and ICAM-1 were among other endothelial cell surface adhesion molecules evoked significantly. Upregulation of the ICAM-1 and VCAM-1 (Figure 2) can lead to the temporary release of intercellular junctions, allowing the pathogen to pass through the endothelial barrier and reach ECM. Genes coding laminin subunits LAMB3 (logFC 3.7) and LAMC2 (logFC 4.35) were upregulated as well (Supplementary Data Sheet 5, slide 1). It is known that various pathogens attach to the laminin to colonize the tissue and migrate through the ECM (Singh et al., 2012).

The junctional molecule CADM3, which is involved in the regulation of function and structure of junctions, was significantly downregulated (logFC −2.18) (Figure 2). A transcript of LIM zinc finger domain 2, involved in the cell junction organization, was also downregulated (LIMS2 logFC −1.57). On the other hand, claudin 1, which is important for maintaining tight junction integrity, was upregulated in challenged cells (CLDN1 logFC 1.93). KDR gene, encoding tyrosine-protein kinase that promotes differentiation of endothelial cells and reorganization of the actin cytoskeleton, was also evoked significantly (logFC 1.57). Transcript of Thrombospondin 1 (THBS1 logFC 2.00) that mediates cell-to-matrix interaction was upregulated along with the transcripts of Angiopoietin-2 (ANGPT2 logFC 1.54) and integrin alpha-2/beta-1 (ITGA2 logFC 1.67).

### Expression of Genes Involved in an Extracellular Matrices Organization

*Borrelia* does not produce any proteases that can digest the ECM, however, it upregulates the host proteases like metalloproteinases. ADAM8, a disintegrin and metalloproteinase domain-containing protein was upregulated in induced hBMECs (logFC 3.89) (Figure 2). Members of the ADAMs family have proteolytic and adhesive functions and may play a role in remodeling the ECM. Simultaneous upregulation of the proteases and the genes encoding structural components of the ECM like FBN1, LAMB3, and LAMC2 is an interesting result observed in this study (Supplementary Data Sheet 5, slide 2). Upregulation of the adhesive molecules related to ECM was found, e.g., CEACAM (logFC 2.00, Figure 2) that plays an important role in the activation of angiogenesis and previously mentioned ICAM-1 and VCAM-1. Induced expression of some of the ECM receptors was also noteworthy in challenged cells, e.g., tyrosine-protein kinase (KDR logFC 1.57) and CD44 (logFC 2.28) (Figure 2).

### Expression of Genes Involved in Vesicle-Mediated Transport

Although *Borrelia* prefers the paracellular way of translocation of BBB, some reports have shown transcellular passage. Vesicle-mediated transport is one of the key processes necessary for transcellular translocation. Thus, in brief, we have presented the deregulation of genes important in this process. DEGs were enriched into two GO biological processes associated with a transcellular passage, namely “membrane trafficking” and “binding and uptake of ligands by scavenger receptors” (Figure 2). In the latter GO process, we found only one evoked gene, the SAA1 (logFC 2.98) that encodes serum amyloid 1. In the biological processes “membrane trafficking” 8 genes (LDLR, SGIP1, GJA1, RALA, SAT1, DAB2, IL7R, and OPTN) were overexpressed, wherein Interleukin-7 receptor (IL7R logFC 3.67) was the most upregulated gene. SH3 domain GRB2 like endophilin interacting protein 1 (SGIP1 logFC 1.69), DAB adaptor protein 2 (DAB2 logFC 2.11), and low-density lipoprotein receptor (LDLR logFC 1.44) were also found upregulated in induced hBMECs (Figure 2), and together with IL7R they belong in the “clathrin-mediated endocytosis” pathway.

### Cellular Stress

**FIGURE 3:**
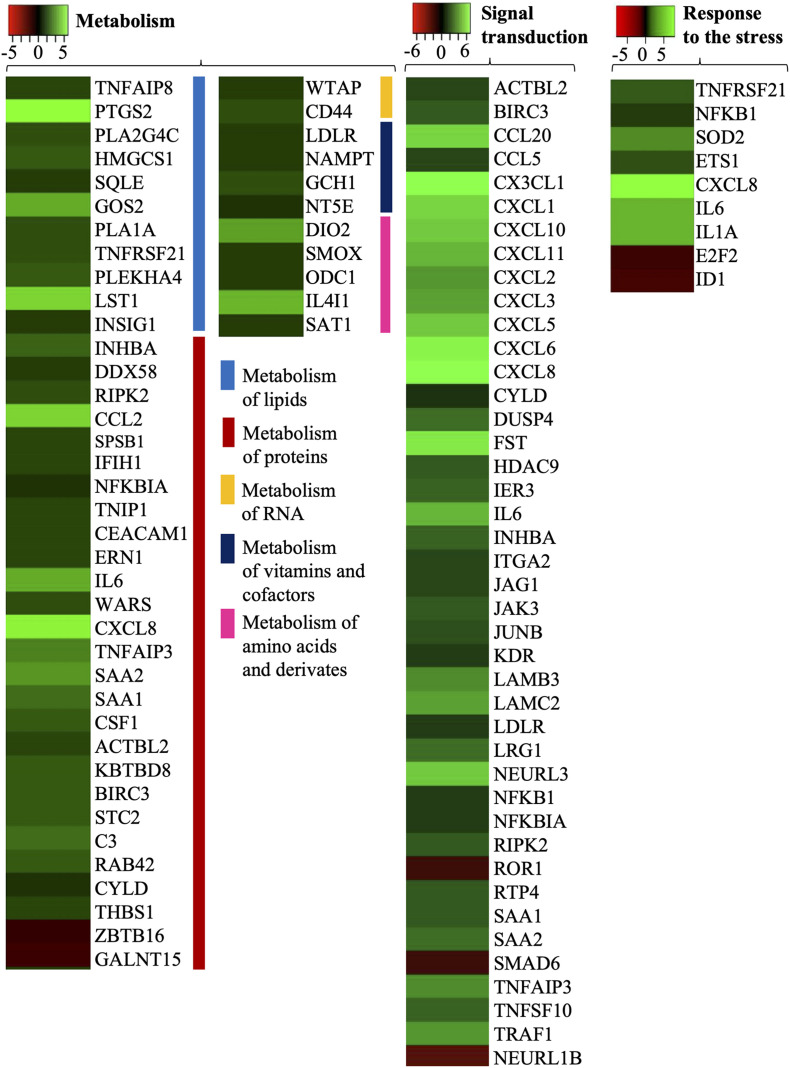
Gene ontology biological processes (response to the stress, metabolism, and signal transduction) activated by *B. bavariensis* in hBMECs. Green shaded genes—upregulated, red shaded genes—downregulated. The intensity of the color indicates the degree of expression level. The range of the fold change (logFC) is presented in the scale.

Cellular stress response includes various changes that influence the cells in response to the external environment, including temperature, toxins, and infection. Several stress-related proteins overlap with immune proteins, thus the cell response in infection may be due to the production of cytokines and chemokines. Nine genes were identified that are involved in “response to the stress” (Figure 3 and Supplementary Data Sheet 3). Among immune related proteins IL8, IL1α, and IL6 were upregulated. An important molecule related to oxidative stress, SOD2 (superoxide dismutase 2) was significantly upregulated (logFC 3.06). Another 3 genes (ID1 logFC −2.04, E2F2 logFC −1.73, ETS1 logFC 2.15) related to the transcriptional regulator of the cell cycle or DNA binding were evoked (Figure 3).

### Metabolism

Cells may change their metabolism in response to an infection. *Borrelia* can affect central metabolic pathways like carbohydrate metabolism, lipid metabolism, and redox metabolism (Kerstholt et al., 2020). In the present study, expression of the genes related to the metabolism of lipids, metabolism of vitamins and cofactors, metabolism of protein and amino acids, and metabolism of RNA was altered significantly (Figure 3), whereas none of the genes in carbohydrates and redox metabolism was evoked. In the case of lipid metabolism, cyclooxygenase (Prostaglandin-Endoperoxide Synthase 2, PTGS2 logFC 7.2), and phospholipases (PLA1A logFC 2.2; PLA2G4C logFC 2.1) were significantly upregulated (Supplementary Data Sheet 3). PTGS2 together with leukocyte-specific transcript 1 protein (LST1, logFC 5.95) were the most upregulated genes in lipid metabolism. Upregulation of two genes (WTAP logFC 1.47, CD44 logFC 2.28) related to RNA metabolism was observed in challenged cells, whereas in the case of metabolism of vitamins and cofactors, four genes were evoked, namely 5′-nucleotidase (NT5E logFC 1.42), GTP cyclohydrolase I (GCH1 logFC 2.09—protect against death cells), NAMPT (logFC 1.59), and LDLR (logFC 1.44). Four genes (DIO2, SMOX, ODC1, and SAT1) related to the metabolism of polyamines and IL4I1 related to the metabolism of phenylalanine and tyrosine (all five related to amino acids metabolism) had altered expression in challenged cells (Supplementary Data Sheet 3). In this study, 27 genes related to the metabolism of proteins were identified, in which 25 were upregulated and 2 were downregulated (ZBTB16 and GALNT15).

### Signal Transduction

Signal transduction includes a process of a physical or chemical signal that is transmitted through a cell, which results in a cellular response. Using Reactome software 42 DEGs involved in the biological process “signal transductions” were identified. Among those DEGs 39 genes were upregulated and 3 genes were downregulated (ROR1 logFC −1.73, SMAD6 logFC −1.65 and NEURL1B logFC −2.6) (Figure 3). Activation of cell receptors triggers signaling cascades that may influence cell differentiation, proliferation, or survival. Signal transduction involves many types of receptors namely receptor tyrosine kinases (RTKs), TGF-β receptors, G-protein coupled receptors (GPCRs), or NOTCH receptors. Six DEGs (LAMC2, JUNB, HDAC9, ITGA2, CXCL6, and LAMB3) related to RTKs were found in the changed cells. RTK-dependent activation of RAF/MAP kinase cascade stimulates the process of gene expression, proliferation, metabolism or differentiation, and apoptosis. Another type of receptor, the TGF-β, transmits extracellular signals by activation of SMAD complex, which plays a role as a transcriptional factor (SMAD6 logFC −1.65). In signaling cascades trigger by GPCRs 12 genes were detected, where CXCL8 (logFC 6.82) was the most upregulated gene, whereas in the case of NOTCH receptor signaling pathway 3 upregulated genes were observed (JAG1, KDR, and NEURL1B, Figure 3).

### Differentially Expressed Genes Related to Immune Response

**FIGURE 4:**
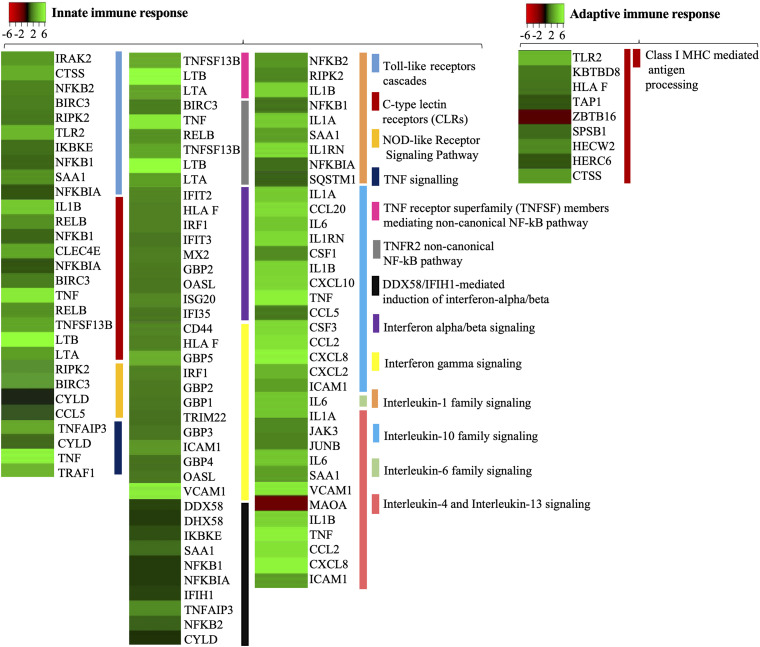
The differentially expressed genes related to the immune response in hBMECs activated by *B. bavariensis*. Heat maps showing biological processes related to the immune response. Green shaded genes—upregulated, red shaded genes—downregulated. The intensity of the color indicates the degree of expression level. The range of the fold change (logFC) is presented in the scale.

It is important to understand the immune response in endothelial cells of brain microvasculature, as the activation of immune pathways like NF-κB or TNF leads to upregulation of adhesion molecules and recruitment of leukocytes at the site of infection, which ultimately help *Borrelia* with stationary adhesion on endothelial cells and cross the BBB. Using the Reactome server we identified 39 DEGs related to the innate immune system and 9 DEGs belonging to the adaptive immune system evoked in induced cells (Figure 4).

#### Receptors on Host Cells (Innate Immune Response)

Pattern recognition receptors (PRR) represent the first line of cell-sensing mechanism, which recognize pathogen-associated molecular patterns (PAMPs). Members of the PRR family, the TLRs, C-type lectin receptors (CLRs), and NOD-like receptors, are expressed not only by the cells of the immune system but also by other cell types. Activation of the members of the TLR family, through bacterial PAMPs, was expected in this study. Interestingly, the expression of TLR4 remained unaltered (logFC 0.079) in the challenged cells. Although *Borrelia* is a Gram-negative bacteria, its atypical outer membrane lacks lipopolysaccharide (Takayama et al., 1987; Kelesidis, 2014), which explains why the TLR4 was not evoked. TLR2, which detects bacterial surface lipoproteins, was upregulated significantly (logFC 4.4, Figure 4 and Supplementary Data Sheet 5, slide 3), while TLR1 recognizes lipoprotein in concert with TLR2 was not evoked (logFC 0.22). The dimer of TLR5 that recognizes flagellin was not detected in the challenged cells. Borrelial flagella are localized in the periplasmic space and not exposed to the bacterial surface, thus it is not sensed by the TLR5.

C-type lectin receptors are expressed on various cell types like endothelial cells, epithelial cells, microglia-like cells, or neurons (Sattler et al., 2012; Suzuki et al., 2013; Xie et al., 2017). CLRs are divided according to the recognition pattern (bacteria, viruses, fungi, or dead cells), while Dectin-1, DC-SIGN, Dectin-2, CLECSF8, CLEC4E (macrophage-inducible C-type lectin; MINCLE) are associated with bacterial PAMPs. Among these PRRs associated with bacterial ligands, only the MINCLE (CLEC4E logFC 3.81) was significantly upregulated by *B. bavariensis*. MINCLE is known to recognize bacterial glycolipids, and it may be predicted that upregulation of MINCLE might be due to borrelial surface glycolipids, e.g., BBGL1, BBGL2. Among identified genes, we also noticed several transcripts encoding C-type lectin proteins (e.g., CLEC11A, CLEC2B, CLEC2D, CLEC14A, and CLEC16A), however, their expression remained at the basal level in challenged cells (logFC −0.29 to 1.11) (Supplementary Data Sheet 2).

NOD-like receptors, expressed on several cell types including endothelial cells (Heim et al., 2019), are divided according to the nature of their N-terminal effector domain (e.g., NOD1, NOD2, NLRC2 NLRC5, NLRP1, or NLRP2), wherein NOD1 and NOD2 are the best-characterized receptors recognizing bacterial PAMPs. Among these receptors, expression of NOD2 was evoked (logFC 4.38, Figure 4) by *B. bavariensis*, while other receptors of the NOD family remained uninduced (NOD1 logFC −0.23; NLRC5 logFC 0.61; and NLRP1 −0.26) (Supplementary Data Sheet 2).

#### Adaptors Molecules (Innate Immune Response)

Induction of TLR2 triggers MyD88-dependent signaling pathway, which leads to the interaction of IRAK kinase family members (IRAK2 and IRAK4) with signal transducer TRAF6, activation of IκB kinase (IKK), translocation of NF-κB into the nucleus, and induction of pro-inflammatory genes. Among these signaling molecules the adaptor molecule MyD88 (logFC 1.3), kinase IRAK2 (logFC 3.39) were upregulated (Supplementary Data Sheet 5, slide 3) while IRAK4 (logFC 0.15) and TRAF6 (logFC 0.43) had a basal expression (Supplementary Data Sheets 2, 3).

Activation of MINCLE triggers the recruitment of SYK that leads to the formation of complex CARD9/MALT1/Bcl10 that can activate transcriptional factors NF-κB and AP1. RNA-seq detected MALT1 (logFC −0.21, Supplementary Data Sheet 2) and Bcl10 (logFC 0.69, Supplementary Data Sheet 2), however, their expression was at the basal level. Transcript CARD9 was not present between detected genes.

Activation of NOD2 receptors also leads to activation of the key transcriptional factors NF-κB and AP1 via activation of IKK mediated by Receptor Interacting Serine/Threonine Kinase 2 (RIPK2). In this study RIPK2 (logFC 2.27) was significantly upregulated. A negative regulator of NOD2 signaling cascade, the CYLD Lysine 63 Deubiquitinase (CYLD), was also detected upregulated in the study (CYLD logFC 1.35, Supplementary Data Sheet 3).

#### NF-κB and Tumor Necrosis Factor (Innate Immune Response)

Signaling pathways triggered by activation of PRRs lead to activation of NF-κB, which in turn activates several transcripts of inflammatory molecules, adhesins, chemokines, and cytokines (Supplementary Data Sheet 5, slide 4). Both subunits of NF-κB, NFKB1 (logFC 1.57) and (NFKB2 logFC 2.59), were significantly upregulated. Consequently, pro-inflammatory molecules (IL1α, IL1β, IL8, CCL2, CXCL6, CXCL1, CCL5, CCL20, CXCL10, CXCL11) were also significantly induced (logFC 1.79–6.82). The IL6, which may act as both pro- or anti-inflammatory, was also upregulated (logFC 4.66, Figure 4). Significant upregulation of anti-inflammatory immune molecules IL24 (logFC 4.96) and IL1RN was also noticed (logFC 5.52). As mentioned above, NF-κB is also responsible for the upregulation of adhesins ICAM-1, VCAM-1, and E-selectin (Figure 4 and Supplementary Data Sheet 3), which can affect cell junctions and the cytoskeletal remodulation.

Tumor necrosis factor (TNF), a pro-inflammatory cytokine, was significantly upregulated (TNF logFC 6.69). TNF can mediate signaling pathways through two receptors TNFR1 and TNFR2, that occur on various cell types, e.g., cells of the immune system, endothelial cells, and epithelial cells (Supplementary Data Sheet 5, slide 5). None of the TNF receptors was detected in this study. The binding of TNF to TNFR1 activates pro-inflammatory signaling pathways mediated via its downstream molecules TRADD, TRAF2, TRAF5, RIPK1, which leads to activation of NF-κB via IKK. In this study, adaptors molecules TRADD, TRAF2, TRAF5, RIPK1, TAB 1/2/3, and TAK1 were detected, however, their expression remained at the basal level (logFC −0.09 to 0.63, Supplementary Data Sheet 2). The binding of TNF to TNFR2 activates anti-inflammatory response, mediated through its adaptor molecules (TRAF1, TRAF2, TRAF3) and IKKs (via MAP3K14, PIK3CD, PIK3CB, PIK3R1, PIK3R3, PIK3R2, and PIK3CA) (Wajant and Siegmund, 2019). From these adaptor molecules, TRAF1 was significantly upregulated (logFC 3.09) while TRAF2 and TRAF3 had basal expression (TRAF2, logFC −0.09; TRAF3, logFC 0.93, Supplementary Data Sheet 2). Several molecules that are responsible for the IKKs activation were detected (MAP3K14, PIK3CB, PIK3CD, PIK3R1, and PIK3R3), however, their expression levels remained at the basal level (logFC −0.09 to 0.93, Supplementary Data Sheet 2).

#### Interferon (Innate Immune Response)

*Borrelia burgdorferi* is known to induce interferon responses (Miller et al., 2008; Krupna-Gaylord et al., 2014). IFNβ balances the expression of pro- and anti-inflammatory molecules in the brain and thus reduces the entry of inflammatory cells across the BBB (Kieseier, 2011). Significant upregulation of the gene coding type I interferon (IFNB1, logFC 4.28) and its regulatory molecule IRF1 (logFC 2.1) was noticed when cells were induced with *B. bavariensis* (Supplementary Data Sheet 5, slide 5 and Figure 4). IFNβ can be induced via DDX58/IFIH1 (RIG-I signaling pathway) or indirectly via TNFR2 activation (Supplementary Data Sheet, slide 5). DDX58 along with downstream molecules in this pathway were also evoked by *B. bavariensis* namely DDX58 (logFC 1.68), IFIT3 (logFC 1.76), IFIH1 (logFC 1.82), whereas expression of IRF7 and TRIM25 remained at the basic level (logFC 0.68 and 0.72, respectively).

#### MHC Class I (Linkage Between Innate and Adaptive Immune Response)

MHC class I is present on almost all nucleated cells; thus, endothelial cells may process the antigen via MHC class I. Nine DEGs related to “MHC class I mediated antigen processing and presentation” were evoked in the treated cells (Supplementary Data Sheet 3 and Figure 4). Among those genes, Cathepsin S (CTSS, logFC 4.05) was one of the most upregulated genes (Figure 4). It is important to note that, while CTSS primarily participates in the digestion of antigenic proteins to the peptides for MHC presentation, it also cleaves ECM proteins like laminin, elastin and fibronectin, and proteoglycans of the basal lamina.

## Discussion

### Deregulation of Cell Adhesion Molecules and Metalloproteases

Neuroinvasive *Borrelia* can cross the BBB and invade the CNS (Kim, 2008). Most of the studies report the paracellular way of translocation, few suggest a transcellular passage (Comstock and Thomas, 1989; Szczepanski et al., 1990; Grab et al., 2005). Nevertheless, adhesion of *Borrelia* to the host cell surface receptors via membrane lipoproteins and activation of downstream signaling cascades is pivotal to translocate *Borrelia* across the endothelial barrier (Antonara et al., 2011). In the present study upregulation of CD44 and CD47 genes encoding cell surface molecules has been noticed (Figure 2). It is well known that CD44 is involved in cell-cell interactions and cell adhesion, and facilitates bacterial adhesion (Goodison et al., 1999; Ponta et al., 2003; Fu et al., 2019). To our knowledge, the role of CD47 during borrelial infection is a newly discovered mechanism of spirochete invasion (Donta et al., 2021). The bacterial binding to the cell surface also upregulates the expression of adhesive molecules like E-selectin (Ebnet et al., 1997) or integrins (Coburn et al., 1998; Hauck et al., 2012). Significant alteration in expression of the genes encoding E-selectin (SELE) and integrin alpha-2 (ITGA2) (Figure 2) in hBMECs suggest that *B. bavariensis* upregulates both molecules and may exploit for stationary adhesion on the endothelium. Overexpression of ICAM-1 and VCAM-1 on endothelial cells results in the transmigration of leukocytes, enhances vascular permeability, and reduces endothelial barrier properties via activation of TNF mediated inflammatory signaling pathways (Matheny et al., 2000; Cerutti and Ridley, 2017).

Along with upregulation of cell adhesion molecules, *Borrelia* is able to induce proteases to digest endothelial cell junctional proteins at the site of attachment and enhance the permeability of BBB (Hu et al., 2001; Grab et al., 2005). Thus, significant upregulation of the MMPs (like MMP-1, 3, 9) and members of the ADAMs family (Figure 2 and Supplementary Data Sheet 3) was expected in the present study. Although the logFC of MMP-1 and MMP-3 were, respectively, 6.33 and 4.68, these genes were not included in the list of DEGs because of logCPM values < 3.0 (Supplementary Data Sheet 2).

### Alteration in Cell Metabolic Pathways

It is interesting to note that *B. burgdorferi* depends on several host’s metabolic pathways for its survival (Kerstholt et al., 2020). Studies describing spirochetes-host interactions have reported the ability of *Borrelia* to alter the metabolic pathways in human cells (Kerstholt et al., 2018, 2020; Fitzgerald et al., 2020). One of the most significantly affected pathways in human monocytes is glutathione metabolism, required for cytokine production (Kerstholt et al., 2018). In this study, several genes related to glutathione metabolism, namely, SMS, GGCT, ANPEP, LAP3, RRM1, GST1, and MGST2 (the latter two are related to glutathione-S-transferases), were identified but no significant alteration in their expression was observed (Supplementary Data Sheet 2). Probably, endothelial cells do not have altered glutathione metabolism after interaction with *B. bavariensis*. Induction of polyamines, nucleotide metabolites, and phospholipid metabolites in the cells challenged by *Borrelia* was also reported (Kerstholt et al., 2018).

The metabolism of fatty acids and eicosanoids is important during borrelial pathogenesis (Fitzgerald et al., 2020; Kerstholt et al., 2020). Eicosanoids are inflammatory mediators, derived from arachidonic acid by cyclooxygenase (COX) and lipoxygenase (LOX) (Buczynski et al., 2009; Stables and Gilroy, 2011). Cyclooxygenase PTGS2 and phospholipases PLA1A, PLA2G4C were significantly upregulated in hBMECs undergoing *B. bavariensis* infection (Figure 3 and Supplementary Data Sheet 3). The phospholipases present in the host lipid membrane are degraded to obtain choline, which is an essential part of the borrelial outer membrane (Titball, 1993). Gene coding lipoxygenase LOX and numerous cytochromes were also found in our study; however, their expression remained at basal levels (Supplementary Data Sheet 2). Metabolism of glycolipids, mainly sphingolipids, was observed to be altered in patients with neuroborreliosis and it was hypothesized as a result of degeneration of the myelin sheaths (Łuczaj et al., 2017). *Borrelia burgdorferi* is also able to incorporate sphingolipids into its membrane through the exchange of lipid rafts with host cells (Garcia Monco et al., 1992). Expression of beta-1,3-galactosyltransferase 4 (B3GALT4) and ST3 beta-galactoside alpha-2,3-sialyltransferase 2 (ST3GAL2), which are involved in the synthesis of complex sphingolipids, remained at the basal level in our study (Supplementary Data Sheet 2).

### Induction of the Pattern Recognition Receptors and Downstream Signaling Cascades

During bacterial infection, PRRs such as TLRs, CLRs, and NOD-like receptors recognize PAMS and mobilize signaling cascades to activate NF-κB (Geijtenbeek and Gringhuis, 2009; Takeuchi and Akira, 2010). It is a well-recognized fact that expression of PRRs is not just limited to cells of the immune system but various cell types that are not related to the immune system also express PRRs (Sattler et al., 2012; Suzuki et al., 2013; Heim et al., 2019). Thus, fourfold overexpression of TLR2, NOD2, and MINCLE indicates active recognition of spirochete ligands by PRRs of hBMECs (Figure 4 and Supplementary Data Sheet 3). Interestingly, the interplay of TLR2 and NOD2 to induce NF-κB-mediated cytokine response against *Borrelia* with a possible penalty of inflammatory-induced pathology has been documented (Oosting et al., 2010). NF-κB activates transcription of chemokines promoting recruitment of leukocytes, and simultaneously, induces genes encoding adhesion molecules (ICAM-1, VCAM-1, and E-selectin), cytokines (IL6, IL8, IL1α, IL1β), and TNF responsible for remodulation of cell cytoskeleton (Collins et al., 1995; Wooten et al., 1996; Middleton et al., 2000; Lawrence, 2009). Based on upregulated chemokines (CCL5 CCL20, CXCL1, CXCL2, CXCL5, and CXCL10), adhesion molecules (ICAM-1, VCAM-1, and E-selectin), and cytokines (IL1α, IL1β, IL6, and TNF) it could also be predicted that endothelial cells recruit leukocytes during the borrelial invasion (Figure 4). Moreover, remodulation of cell cytoskeleton can benefit borrelial or leukocytes traversal through the endothelial barrier (Carlos and Harlan, 1994; Collins et al., 1995). It is also important to note that in some conditions overexpression of TNF can give rise to vascular injury, followed by endothelial barrier dysfunction (Madge and Pober, 2001). Upregulated IL8 (CXCL8) and IL6 also affect the BBB integrity. Upregulation of IL8 helps to recruit neutrophils to the site of infection, which are involved in the reorganization of ECM and basement membrane (Singer and Sansonetti, 2004; de Oliveira et al., 2013; Jennings and Knaus, 2014), whereas IL6 increases endothelial permeability due to the reorganization of actin filaments (Maruo et al., 1992).

### Non-coding RNAs

RNA-seq in contrast to microarray can identify non-protein coding genes. It is noteworthy that the role of non-coding RNA in bacterial infection is still poorly understood. In this study, five lncRNAs (LINC02015, LINC00520, MIR22HG, ENSG00000271646, ENSG00000270607), three anti-sense RNA (USP50-AS1, ENSG00000238045, ENSG00000272269), and a small Cajal body-specific RNA (SCARNA22) were significantly evoked in challenged hBMECs (Supplementary Data Sheet 3). Besides downregulated SCARN22 (logFC −1.96), all other genes were upregulated, where long intergenic non-protein coding RNA 2015 (LINC02015, logFC 5.69) was the most significantly upregulated. Long non-coding RNAs (lncRNAs) have been demonstrated to play an important role in gene expression, while it is predicted that lncRNAs are helping the host cells against pathogen invasion (Wen et al., 2020). It is also reviewed (Wen et al., 2020) that pathogens can manipulate the host signaling pathways by regulating the host lncRNAs for immune evasion. Some lncRNA are used as a potential diagnostic marker of infection, e.g., MIR3954 HG V1 and MIR3954HG V2 are used as markers for tuberculosis (Yang X. et al., 2016) and LINC00152 in *H. pylori* infection (Yang T. et al., 2016). Expression of three anti-sense RNAs (USP50-AS1, ENSG00000238045, ENSG00000272269) was evoked in our study. These RNAs belong to the small regulatory RNA family comprised of small intergenic sRNAs, intragenic sRNAs, and anti-sense sRNAs (Lybecker and Samuels, 2017). Small regulatory RNAs (sRNA) have emerged in the last decade as important regulators of gene expression via base-pairing target mRNAs (Waters and Storz, 2009; Beisel and Storz, 2010; Storz et al., 2011; Caldelari et al., 2013). In addition, some sRNAs can regulate cellular processes via binding and sequestering target proteins (Lybecker and Samuels, 2017). The importance of the anti-sense RNAs in borrelial infection is still an unexplored area of research.

Although RNA-seq has provided us information on non-coding RNAs, it failed to provide an insight into mRNA splicing variants due to the use of 3′ mRNA-seq library preparation kit (Lexogen) that allows generation of Illumina compatible libraries of sequences close (150–350) not only to the 3′ end of polyadenylated RNA. This limitation can be avoided by combination of 3′ mRNA-seq forward and reverse kits from Lexogen or using long-read technology (e.g., Nanopore).

In summary, this study has revealed upregulation of several adhesins and CD molecules that help stationary adhesion of *Borrelia* on endothelial cells of brain microvasculature. It has shown upregulation of several proteases and other components of ECM, which may compromise the integrity of the tight junction and remodulate the cytoskeleton. Further, it has revealed a plethora of the genes related to cytokines, chemokines, interferon, and TNF that may cause alteration in the BBB integrity. A study has also unfolded the upregulation of genes in biological processes like cellular metabolism and cellular stress that can be exploited by *B. bavariensis.* We believe that systemic dissection of signaling events, revealed by RNA-seq based transcriptome analysis, will help researchers to understand underlying molecular processes that take place during adhesion of *B. bavariensis* on hBMECs and crossing of BBB.

## Data Availability Statement

The datasets presented in this study can be found in online repositories. The names of the repository/repositories and accession number(s) can be found in the article/Supplementary Material.

## Author Contributions

MB and KB conceived the project. KB, JH, and PP performed hBMEC cells culture and the challenge. ZT and EM carried out the RNA isolation and library preparation. KB and ZT designed the Primers for qPCR and performed the qPCR. ZT, AK, and MB performed the bioinformatics analysis and wrote the manuscript. All authors read and approved the final manuscript.

## Conflict of Interest

The authors declare that the research was conducted in the absence of any commercial or financial relationships that could be construed as a potential conflict of interest.

## Publisher’s Note

All claims expressed in this article are solely those of the authors and do not necessarily represent those of their affiliated organizations, or those of the publisher, the editors and the reviewers. Any product that may be evaluated in this article, or claim that may be made by its manufacturer, is not guaranteed or endorsed by the publisher.

## Footnotes

1
https://www.ebi.ac.uk/arrayexpress/

2
https://reactome.org

3
http://www.heatmapper.ca/expression/

4
https://www.genome.jp/kegg/

5
http://www.endmemo.com/algebra/log2.php

6
https://www.socscistatistics.com/tests/pearson/Default2.aspx
