# Supplementary material for: Comprehensive Mapping of the Cell Response to Borrelia bavariensis in the Brain Microvascular Endothelial Cells in vitro Using RNA-Seq
Source: Front Microbiol. 2021 Nov 8;12:760627. doi: 10.3389/fmicb.2021.760627 (PMC8606740; doi:10.3389/fmicb.2021.760627)
Supplement: Supplementary file 1 [file Data_Sheet_1.pdf]

Comprehensive mapping of the cell response to *Borrelia bavariensis* in the brain microvascular endothelial cells *in vitro* using RNA-seq

Zuzana Tkáčová, Katarína Bhide, Evelina Mochnáčová, Patrícia Petroušková, Jana Hruškovicová, Amod Kulkarni, Mangesh Bhide

SUPPLEMENTARY TABLES

Supplementary Table S1. DEGs selected for primers design for qPCR

GENE NAME	Forward primer	Tm of forward primer	Reverse primer	Tm of reverse primer	Annealing temperature	Amplification efficiency
CCL2 (C-C motif chemokine ligand 2)	CAATCAATGCCCCAGTCACCT	60.6 °C	TCCTGAACCCACTTCTGCTTG	60.2 °C	55 °C	97%
CXCL10 (C-X-C motif chemokine ligand 10)	CTGCCTCCCCATATTCCTCG	59.7 °C	TCGGGTGACAAAGACGACTG	60 °C	55 °C	94%
ICAM-1 (Intercellular adhesion molecule 1)	CCGGCCAGCTTATACACAAGA	60.1 °C	CACATTGGAGTCTGCTGGGAA	60.3 °C	55 °C	98%
IL1α (Interleukin 1 alpha)	GACTGCCCAAGATGAAGACCA	50 °C	TTGGATGGGCAACTGATGTGA	59.9 °C	55 °C	95%
TNF (Tumor necrosis factor)	CCTGTAGCCCATGTTGTAGCA	60 °C	GGACCTGGGAGTAGATGAGGT	60 °C	55 °C	80%
VCAM-1 (Vascular cell adhesion molecule 1)	CCCTGAGCCCTGTGAGTTTT	59.9 °C	GGCCACCACTCATCTCGATT	59.8 °C	55 °C	98%
IL 6 (Interleukin 6)	AAAACAACCTGAACCTTCCA	55.3 °C	CTCCAAAAGACCAGTGATGA	55 °C	50 °C	97%
CXCL 1 (C-X-C motif chemokine ligand 1)	AGTGTGAACGTGAAGTCCCC	59.9 °C	ATGGGGGATGCAGGATTGAG	59.5 °C	55 °C	94%
CXCL 11 (C-X-C motif chemokine ligand 11)	CCCTGGGGTAAAAGCAGTGA	59.6 °C	GCCTTGCTTGCTTCGATTTG	58.7 °C	55 °C	97%
SELE (Selectin E)	TGTGGAATGATGAGAGGTGCA	59.4 °C	TGAAGCCAGGGTCACACTTG	60.2 °C	55 °C	94% (non-specific amplification)
IL8-CXCL 8 (Interleukin 8, C-X-C motif chemokine ligand 8)	CTCCAAACCTTTCCACCCCA	59.8 °C	TTCTCCACAACCCTCTGCAC	59.9 °C	55 °C	98%
SLC16A12 (solute carrier family 16 member 12)	ATTGTGGCTGGCTGTTTCCT	60 °C	CCATGCCGTTTGTGCGTAAT	60 °C	55 °C	93%
METTL7A (methyltransferase like 7A)	GGTGCTGTGCTCTGTGAAGA	60 °C	GGATCCAGGACTTGTTGCCA	60 °C	55 °C	92%
TSC22D3 (TSC22 domain family member 3)	GACAACAAGATCGAACAGGCC	60 °C	TTCTCCACCAGCTCTCGGAT	60 °C	55 °C	99%
TIMP4 (TIMP metallopeptidase inhibitor 4)	ACGCCTTTTGACTCTTCCCT	60 °C	AGGGCTCGATGTAGTTGCAC	60 °C	55 °C	93%
SEMA6C (semaphorin 6C)	GACAGTGCTGAAGGTGCTGA	60 °C	AAAGCCTGTGACCCTCAGTG	60 °C	55 °C	93%
NPR3 (natriuretic peptide receptor 3)	ACCTGAGTTTGAGAAGTTTTCCATG	60 °C	AGACGTAGAGGAGGATGGCA	60 °C	55 °C	93%
MAOA (monoamine oxidase A)	GTCTGCCCTGTGGTTCTTGT	60 °C	CGCTCACTTGACCAGATCCA	60 °C	55 °C	97%
WSCD1 (WSC domain containing 1)	GGGGCTGGAAGAGTGTAACC	60 °C	CTGGGGAAGGCATGTGTGAT	60 °C	55 °C	93% (non-specific amplification)

ATOH8 (atonal bHLH transcription factor 8)	CCTCCGAGATCAAAGCCCTG	60 °C	CCCATATGAGTAGCACGGCA	60 °C	55 °C	94% (non-specific amplification)
CADM3 (cell adhesion molecule 3)	ACGGGAAAAAGACACAGCCA	60 °C	TGTATGCGGGTTGGTTCTCC	60 °C	55 °C	98%
NFIA (nuclear factor I A)	CAGGAAGTGGCAGTCAGTCA	60 °C	GAGGAGGTCTGTGAAGGGGA	60 °C	55 °C	98%
CCL20 (C-C motif chemokine ligand 20)	AGCAAGCAACTTTGACTGCTG	59.9 °C	TTGGATTTGCGCACACAGAC	59.7 °C	55 °C	94% (non-specific amplification)
LTB (lymphotoxin beta)	CTCCAGGGGAGGGGTTCC	60.7 °C	AGTCCTCCCTGATCCTGGG	59.7 °C	55 °C	86%
SAA2 (serum amyloid A2)	CCTTGGTCCTGAGTGTCAGC	60.3 °C	CATAGTTCCCCCGAGCATGG	60.3 °C	55 °C	92% (non-specific amplification)
NFKB1 (Nuclear factor kappa B subunit 1)	TGGTGGAGTCTGGGAAGGAT	59.9 °C	TCCGAAGCTGGACAAACACA	59,8 °C	55 °C	94%
IL1α (Interleukin 1 alpha)	GACTGCCCAAGATGAAGACCA	50 °C	TTGGATGGGCAACTGATGTGA	59.9 °C	50 °C	95%
CD44 (Cluster of Differentiation 44)	CACACCCTCCCCTCATTCAC	60 °C	TCCCTCATGCCATCTGTTGC	60.4 °C	55 °C	93%
CTSS (Cathepsin S)	AAAGGCCCAGTGTCTGTTGG	60.5 °C	AGCCAACCACAAGTACACCA	59.5 °C	55 °C	95%
IL1B (Interleukin 1 beta)	GTGTTCTCCATGTCCTTTGT	55.6 °C	TTTGGGATCTACACTCTCCA	55.2 °C	50 °C	95%
SAA1 (serum amyloid A1)	TTTTGATGGGGCTCGGGAC	60 °C	TCGGTGATCACTTCTGCAGC	60.4 °C	55 °C	92% (non-specific amplification)

Best 10 primer pairs were selected (colored green in table) based on amplification efficiency and specific amplification judged by high resolution melting curve.

Supplementary Table S2. Primers used in qPCR validation

Protein (Gene)	Sequence used to design primers	Primer name	Sequence (5´-3´)	Amplicon length (bp)	Annealing temperature
**primers used for qPCR**					
Intercellular adhesion molecule 1 (ICAM-1)	BT006854.1	ICAM-1F	CCGGCCAGCTTATACACAAGA	124	60°C
		ICAM-1R	CACATTGGAGTCTGCTGGGAA		
Vascular cell adhesion molecule 1 (VCAM-1)	AK291732.1	VCAM-1F	CCCTGAGCCCTGTGAGTTTT	138	60°C
		VCAM-1R	GGCCACCACTCATCTCGATT		
C-C motif chemokine ligand 2 (CCL2)	X14768.1	CCL2F	CAATCAATGCCCCAGTCACCT	175	55°C
		CCL2R	TCCTGAACCCACTTCTGCTTG		
C-X-C motif chemokine ligand 11) (CXCL11)	Y15220.1	CXCL11F	CCCTGGGGTAAAAGCAGTGA	149	60°C
		CXCL11R	GCCTTGCTTGCTTCGATTTG		
Interleukin 6 (IL6)	M18403.1	IL6F	AAAACAACCTGAACCTTCCA	104	55°C
		IL6R	CTCCAAAAGACCAGTGATGA		
Interleukin 8, C-X-C motif chemokine ligand 8 (IL8)	BT007067.1	IL8F	CTCCAAACCTTTCCACCCCA	153	55°C
		IL8R	TTCTCCACAACCCTCTGCAC		
TSC22 domain family member 3 (TSC22D3)	AF153603.1	TSC22D3F	GACAACAAGATCGAACAGGCC	107	60°C
		TSC22D3R	TTCTCCACCAGCTCTCGGAT		
Monoamine oxidase A (MAOA)	AK293926.1	MAOAF	GTCTGCCCTGTGGTTCTTGT	116	60°C
		MAOAR	CGCTCACTTGACCAGATCCA		
Cell adhesion molecule 3	AY358332.1	CADM3F	ACGGGAAAAAGACACAGCCA	114	60°C

(CADM3)		CADM3R	TGTATGCGGGTTGGTTCTCC		
Nuclear factor I A (NFIA)	U07809.1	NFIAF	CAGGAAGTGGCAGTCAGTCA	121	60°C
		NFIAR	GAGGAGGTCTGTGAAGGGGA		
β–microglobulin (B2M)	XR_002957658.1	B2MF	GCTCGCGCTACTCTCTCTTT	134	55°C
		B2MR	CGGATGGATGAAACCCAGACA		

Supplementary Table S3. Total number of detected clusters and mapped genes

Sample		Total number of detected clusters/raw reads	Total number of filtered genes
Borrelia bavariensis	1	13170882	11 398
	2	12387003	
	3	13420391	
Negative control	1	10185750	11 398
	2	11548712	
	3	10507851	

1, 2 ,3 present biological replicates

SUPPLEMENTARY FIGURES

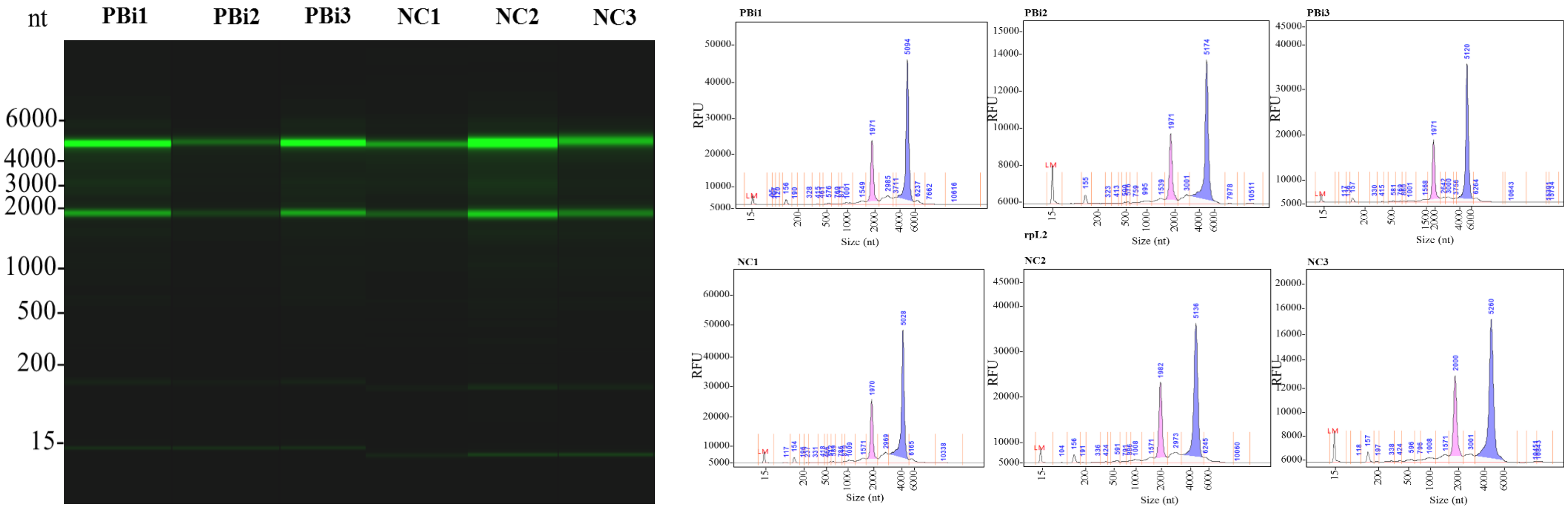

Supplementary Figure S1. Upper figure (Electrophoresis) - assessment of integrity of RNA isolated from hBMECs incubated with *B. bavariensis* (PBi1, 2, 3) and without any protein (negative control, NC1, 2, 3). **Graphs on the right hand (PBi1 to NC3) -** graphical representation of peaks of RNA derived from electrophoresis. RFU – relative fluorescence unit

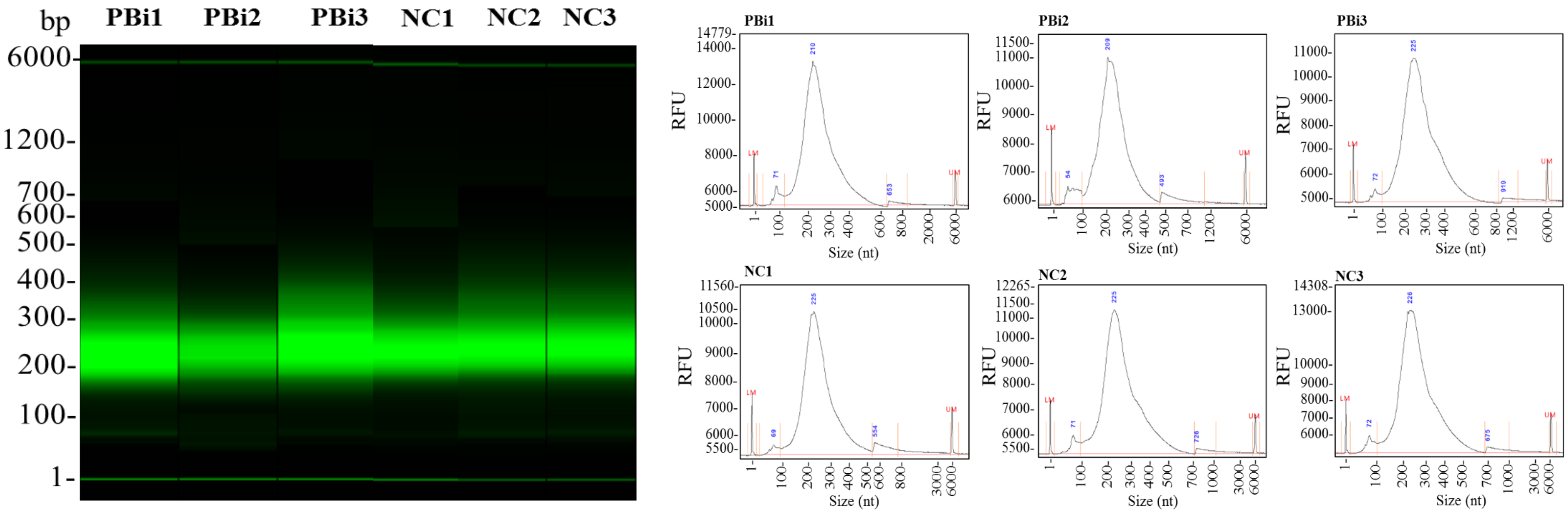

Supplmentary Figure S2. Upper figure (Electrophoresis) - Quality control of libraries prepared with QuantSeq 3' mRNA kit used for sequencing. hBMECs induced with *B. bavariensis* (PBi1, 2, 3*)* and without any protein (negative control, NC1, 2, 3). Note that the ideal fragment size should be between 150-300 bp. **Graphs on the right hand (PBi1 to NC3)** - graphical representation of the peaks of DNA fragments derived from electrophoresis. RFU – relative fluorescence unit.

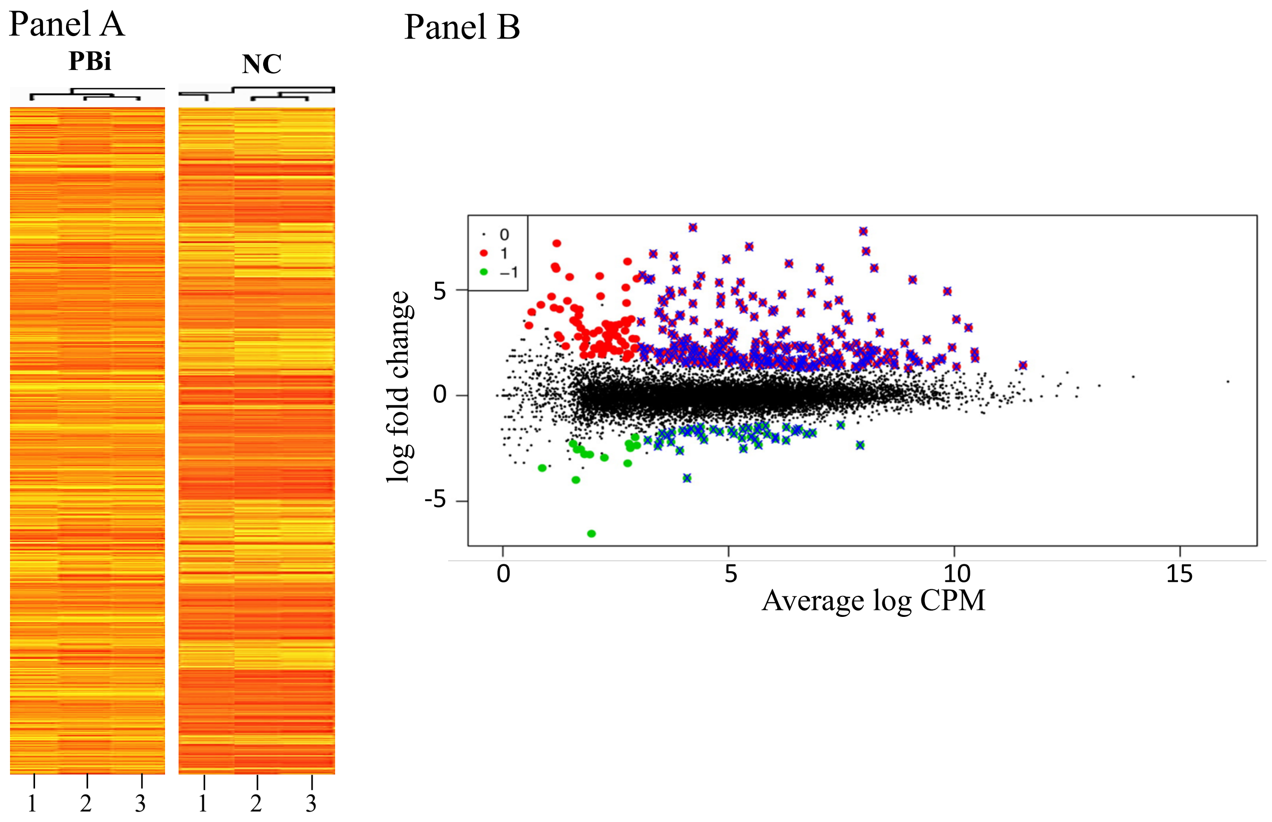

Supplementary Figure S3. Panel A. Heat map generated from sequence reads (1, 2, 3 represents triplicates). **Panel B.** A scatter plot showing genes (each dot presents a single gene). Log fold change with +/- 1.2 was used to select DEGs. All genes which are within this range are presented with black dots (non significant). A cutoff of 3 for average log CPM was set. All the genes more than this cutoff were selected as DEGs and presented with blue cross.
