# Supplementary material for: Comprehensive Mapping of the Cell Response to Borrelia bavariensis in the Brain Microvascular Endothelial Cells in vitro Using RNA-Seq
Source: Front Microbiol. 2021 Nov 8;12:760627. doi: 10.3389/fmicb.2021.760627 (PMC8606740; doi:10.3389/fmicb.2021.760627)
Supplement: Supplementary file 5 [file Data_Sheet_5.PDF]

The schematic view of the events in the ECM-Receptor interaction in the hBMECs induced with *B. bavariensis* (Slide 1).

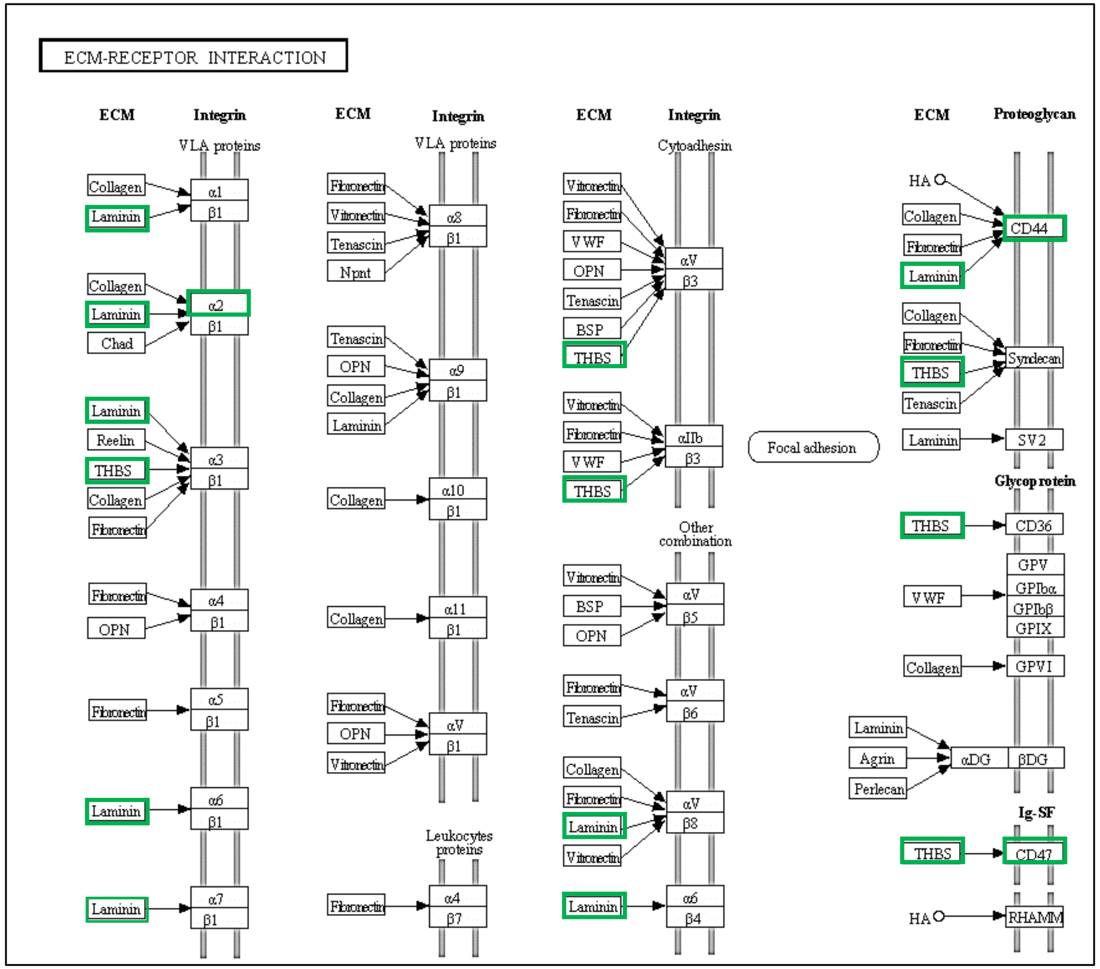

Green boxes – upregulated genes; red boxes – downregulated genes
Pathways are downloaded from the “KEGG“ (Kyoto Encyclopedia of Genes and Genomes).

The schematic view of the events focal adhesion in the hBMECs induced with *B. bavariensis* (Slide 2).

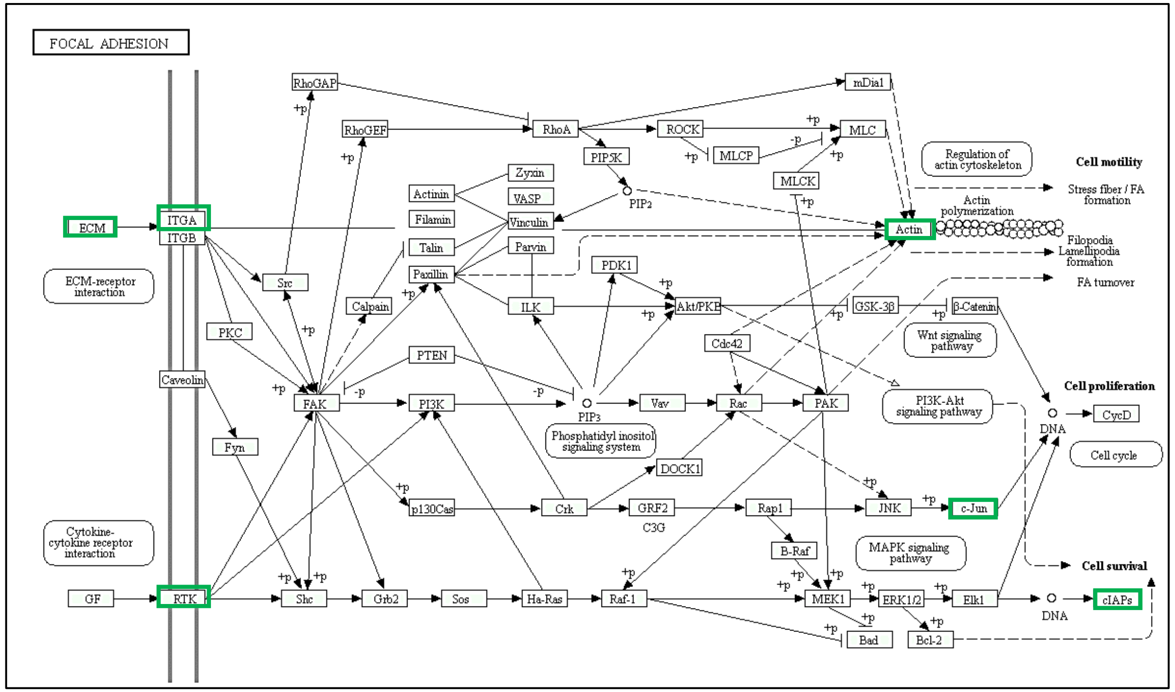

Green boxes – upregulated genes; red boxes – downregulated genes
Pathways are downloaded from the “KEGG“ (Kyoto Encyclopedia of Genes and Genomes).

The schematic view of the events in Toll-like receptor signaling pathway in the hBMECs induced with *B. bavariensis* (Slide 3).

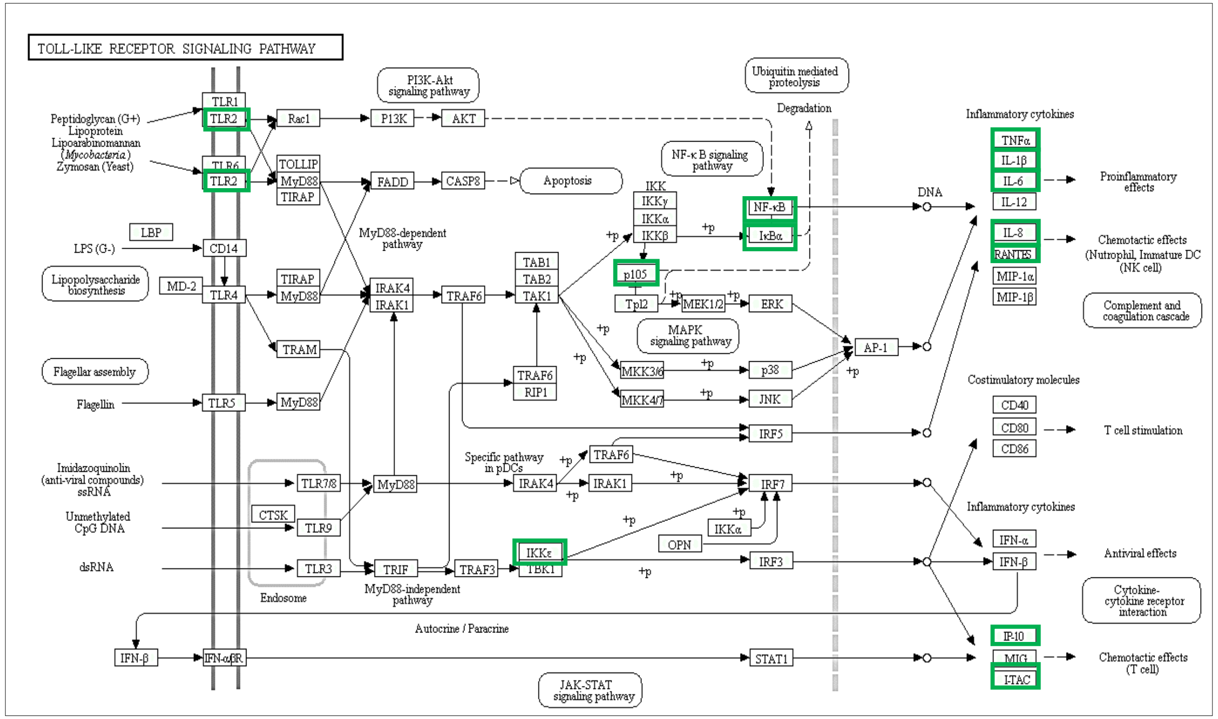

Green boxes – upregulated genes; red boxes – downregulated genes

Pathways are downloaded from the “KEGG“ (Kyoto Encyclopedia of Genes and Genomes).

p105-NFKB1, RANTES-CCL5, I-TAC-CXCL11

The schematic view of the events in NF-KAPPA B signaling pathway in the hBMECs induced with *B. bavariensis* (Slide 4).

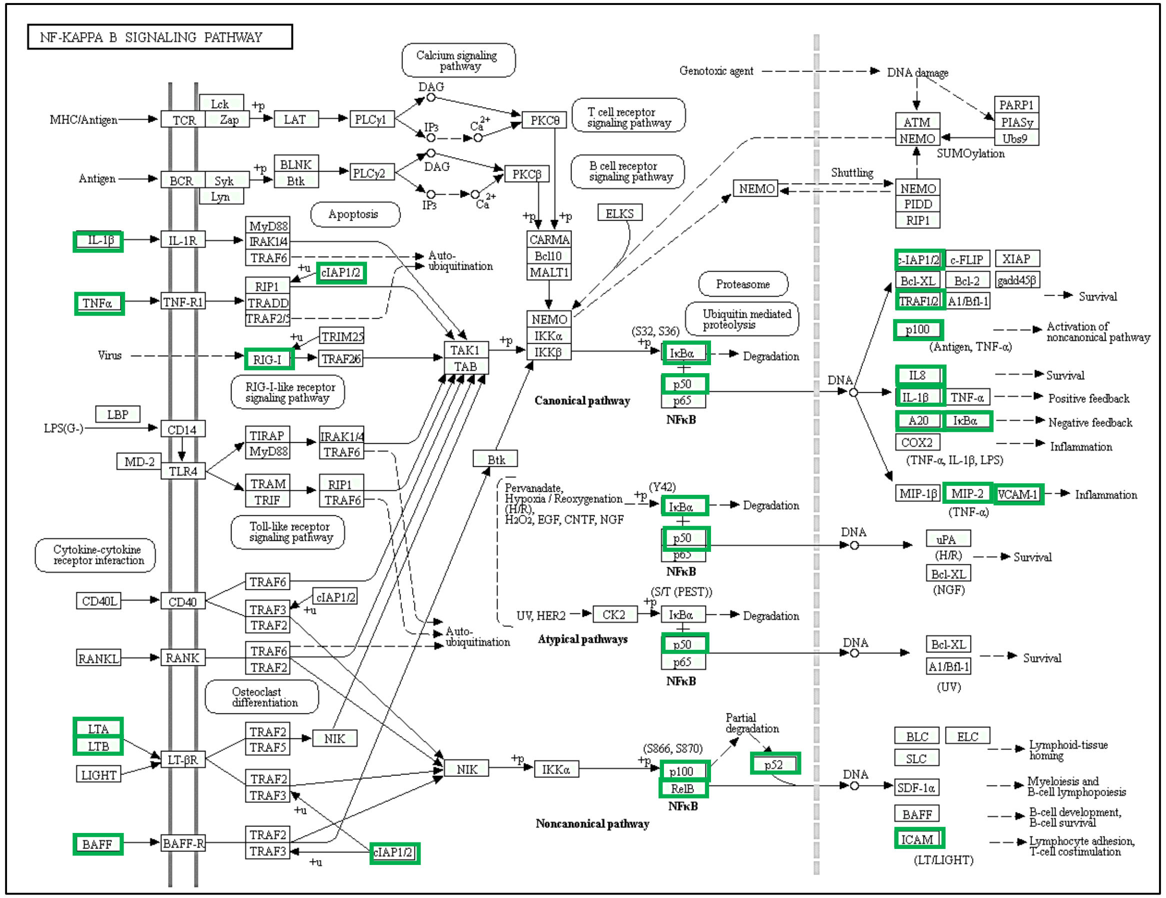

Green boxes – upregulated genes; red boxes – downregulated genes

Pathways are downloaded from the “KEGG“ (Kyoto Encyclopedia of Genes and Genomes).

BAFF-TNFSF13B, p50-NFKB1, p100-NFKB2, MIP-2-CXCL1, A20-TNFAIP3

The schematic view of the events in the TNF signaling pathway in the hBMECs induced with *B. bavariensis* (Slide 5).

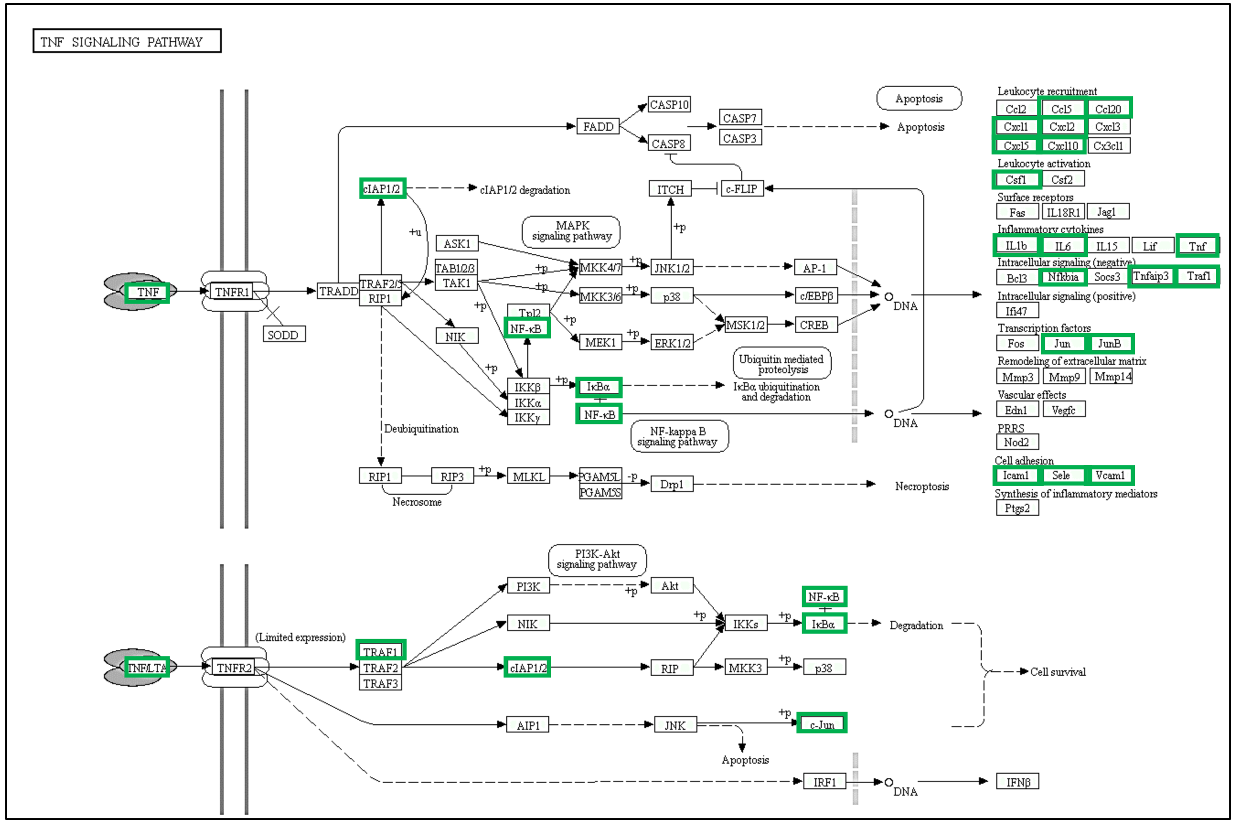

Green boxes – upregulated genes; red boxes – downregulated genes
Pathways are downloaded from the “KEGG“ (Kyoto Encyclopedia of Genes and Genomes).
